# A Wrist-Inspired Magneto-Pneumatic Hybrid-Driven Soft Actuator with Bidirectional Torsion

**DOI:** 10.34133/cbsystems.0111

**Published:** 2024-03-28

**Authors:** Yan Xu, Kaiwen Ju, Chao Zhang

**Affiliations:** ^1^ School of Aeronautics and Astronautics, Zhejiang University, Hangzhou, Zhejiang 310027, China.; ^2^ Huanjiang Laboratory, Zhuji, Zhejiang 311800, China.

## Abstract

A novel wrist-inspired soft actuator, which is driven by a magneto-pneumatic hybrid system and based on a Kresling origami unit, is proposed. The geometric model, kinematic analysis model, and quasistatic analysis model of the Kresling origami unit are presented. A key focus is on the formulation and investigation of the variation in rotation angle using the kinematic analysis model. A wrist-inspired soft actuator is designed, and its quasistatic characteristics are validated through various experiments. The paper proposes an innovative magneto-pneumatic hybrid actuation method, capable of achieving bidirectional torsion. This actuation method is experimentally validated, demonstrating the actuator's ability to maintain 3 steady states and its capability for bidirectional torsion deformation. Furthermore, the paper highlights the potential of the Kresling origami unit in designing soft actuators capable of achieving large rotation angles. For instance, an actuator with 6 sides (*n* = 6) is shown to achieve a rotation angle of 239.5°, and its rotation ratio exceeds 277°, about twice the largest one reported in other literature. The actuator offers a practical and effective solution for bidirectional torsion deformation in soft robotic applications.

## Introduction

The ability of the human wrist to rotate around the forearm axis in 2 directions is crucial for many daily activities. This rotation, limited to a range of approximately [−90°, 90°], restricts the wrist’s capacity to execute complex operational tasks. For example, when we open or lock a door with a key, our wrist performs a large rotational movement. When we screw, the wrist needs to twist 180° several times. However, due to the limited rotation angle, the hand needs to leave the key or screwdriver several times to complete the entire work process. In addition, the wrist often needs to perform bidirectional torsion actions. For example, in some chemical experiments, we shake test tubes or other instruments by repeatedly rotating the wrist in 2 directions around the initial position. Similarly, the human neck’s ability to twist bidirectionally provides a wide range of vision. The dexterity in manipulating objects comes from the human wrist’s capacity for bidirectional twisting. The bidirectional torsion motion will enhance the soft robot’s flexibility and maneuverability. In the context of robotics, especially in designing soft robotic arms and humanoid robots, replicating and enhancing this bidirectional torsion ability is a important challenge. The development of a torsion actuator capable of large, bidirectional torsion movements is a key focus in the field. The creation of an actuator with superior torsion motion capabilities compared to the human wrist not only is a technological advancement but also holds promising application prospects in various complex operational tasks in soft robotics.

The deformation modes of soft actuators include stretching, contraction, bending, twisting, and combinations of them. Compared with contraction and bending actuators, the design and manufacture of twisting actuators are more challenging and difficult. To date, some twisting actuators have been proposed that are actuated by different external stimuli, including pneumatic [1–6], magnetic [7], and temperature [8,9] stimuli. A twisting motion can be achieved by material selection, structure design and manufacture, and driving control of the actuators. Pneumatic twisting actuators include inflate-drive actuators and vacuum-driven actuators. Schaffner et al. [2] reported a 3-dimensional (3D) printing platform for the seamless digital fabrication of pneumatic twisting actuators, which comprise an elastomeric body whose surface is decorated with reinforcing stripes at a well-defined lead angle. By optimizing the fiber angle, the actuators achieved a rotation angle up to 225°. Yang et al. [5] demonstrated a single-unit buckling actuator in which elastic beams bend and buckle in ways that produce a twisting motion when negative pressure is applied, which could perform a rotation angle of 30°. Wu et al. [7] introduced a magnetically controlled actuator based on Kresling patterns to achieve a maximum rotation angle 120° through precise magnetic actuation. Shim et al. introduced a smart soft composite torsional actuator using a single shape memory alloy (SMA) wire. SMAs are a group of metallic alloys that are able to undergo large reversible deformations under loading/thermal cycles and are able to generate high thermal–mechanical driving forces. The torsional actuator twists by applying an electric current upon Joule heating of the SMA wire and the maximum rotation angle reached 100° [8]. A new type of stimuli-responsive artificial muscle using liquid crystal elastomers (LCEs) is proposed for soft flexible wrists [9], which could perform a rotation angle of 18°. LCEs are composed of polymer network and liquid crystal mesogens. This material can change between a nematic phase and an isotropic phase when subjected to external stimuli, resulting in a macroscopic shape change. The current limitations in the maximum rotation angle of existing actuators underscore the need to design a twisting actuator with a greater capacity for torsion motion. Theoretically, superposition of multiple actuators via series connection provides an arbitrary rotation angle for torsion motion. However, this approach has drawbacks, as it tends to increase the overall size and response time of the system, making it less practical for certain applications. Realizing large rotation ratio in a single actuator remains an unconquered challenge.

Origami is a traditional paper folding technique, which has been used to design soft robots in recent decades. Besides other known origami patterns, Kresling [10] presents the “Kresling pattern” as the natural result of twisting a paper cylinder until it buckles. Due to the natural coupling between axial deformation and rotation, Kresling origami has attracted extensive attention thanks to its unique properties. Kresling origami was used to construct mechanical bit memory switches [11,12], wave guides [13,14], selectively collapsible structures [15], vibration isolators [16], mechanical digital memory [17], and digital computing. There have been some attempts to design twisting actuators and robots using Kresling origami [18–25]. These twisting actuators are actuated by different external stimuli, including motor-tendon [19], pneumatic [21–25], magnetic [7], etc. The manufacturing approach of Kresling origami actuators includes multilayer bonding [21], 3D printing [23], and mold casting [24]. However, for these Kresling twisting actuators, the direction of torsion motion is fixed after the crease design is designed. These twisting actuators always result in unidirectional torsion motion, which cannot vividly produce bidirectional torsion motion. The development of bidirectional torsion motion with a large rotation ratio is therefore desired.

**Fig. 1.**
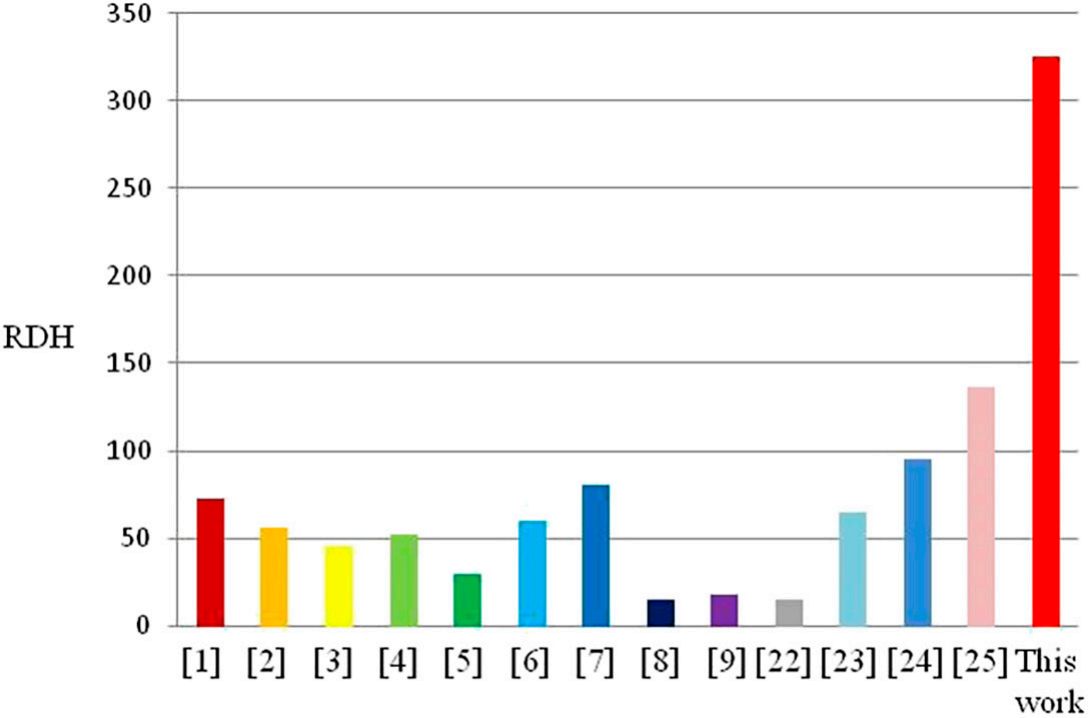
Comparison of rotation ratios RDH of the twisting actuators.

In this work, by designing geometric parameters and a hybrid driving method, a wrist-inspired soft actuator based on a single Kresling origami unit is proposed. The unique design of this actuator allows it to achieve a large rotation angle through bidirectional torsion motion using just a single module. Since the size of the actuators directly influences the rotation angle, to ensure a fair and standardized comparison across different actuators, the rotation angle is normalized by the diameter of section and height of the actuators, which is defined as rotation ratio RDH (= rotation angle * diameter of section / height). The rotation ratio RDHs of this actuator and exiting twisting actuators are compared in Fig. 1. The remainder of this paper is organized as follows: The geometric model, kinematic analysis model and quasistatic analysis model of the Kresling origami unit are presented in the “Kresling Origami Unit” section. The variation in the rotation angle is formulated and investigated by a kinematic analysis model. A wrist-inspired soft actuator is designed and analyzed in the “Wrist-Inspired Soft Actuator” section. The magneto-pneumatic hybrid driving method is proposed for bidirectional torsion. A model is manufactured and used in an experiment to verify the performance of the soft actuator. The design space of a large rotation angle is discussed in Discussion. Finally, Conclusion summarizes the concluding remarks and suggestions for future work.

## Materials and Methods

### Geometric model of Kresling origami unit

**Fig. 2.**
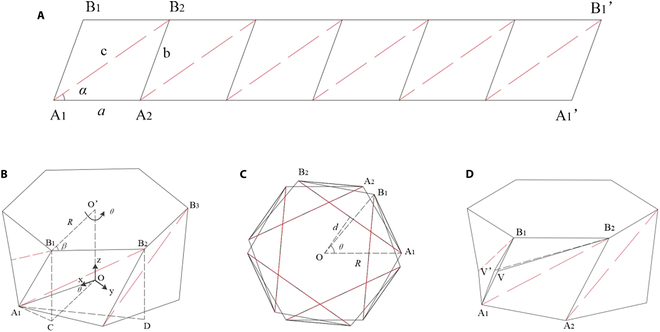
Geometry of the Kresling origami unit. (A) shows the crease pattern for the Kresling origami unit shown in (B), which is derived from the Kresling pattern. Mountain creases are shown as solid lines; black lines and valley creases are shown as dashed red lines. The origami unit configuration can be determined by 4 parameters *n*, *a*, *c*, and *α*. (B) is a Kresling origami unit, which is constructed from the crease pattern in (A). Also shown is the rotation angle *θ* for the twisting motion of the top polygon about the dashed axis. (C) shows a top-down view of the Kresling origami unit shown in (B) while it is completely folded. (D) shows that a “virtual fold” B_2_V can be created between 2 vertices A_1_, B_1_ across a bending panel A_1_B_1_B_2_.

A Kresling origami unit is built by taking a flat flexible plate and dividing it into *n* parallelograms, which are each bisected into 2 similar triangles (Fig. 2A). These triangles form the faces that define the Kresling origami unit. A panel is typically defined by its 2 sides *a*, c and an angle *α*. Therefore, the origami unit can be uniquely defined by the 4 parameters *n*, *a*, *c*, and *α*. From these parameters, the remaining fold lengths and angles are easily determined geometrically from the generic crease pattern and basic polygon relations. By folding along mountain creases and valley creases and then rolling them into a polygonal prism such that points A1 and B1 overlap with A1′ and B1′, the construction of a laterally closed structure is formed, with the panels creating the sidewalls of the unit (Fig. 2B). The bases of the panels, i.e., side *a*, become the *n*-sided top and bottom polygon planes of the Kresling origami unit.

The length *b* of the third side A_1_B_2_ in the triangle A_1_A_2_B_2_ and the radius of the outer circle of the polygon plane are expressed as:

$$b={\left(c^{2}+a^{2}-2\mathrm{ac}\text{cos}\alpha \right)}^{1/2},R=\frac{a}{2\text{cos}\left(\frac{\pi (n-2)}{2n}\right)}$$ (1)

When the origami unit is constructed from the crease pattern into the laterally closed structure (Fig. 1B), all angles and lengths of the triangles remain invariant. Its configuration is expressed by its height *h* and the rotation angle *θ*. The initial 3D configuration of the unit needs to be derived. For vacuum-driven actuators, the initial rotation angle *θ*_0_ of the unit is the smaller solution *θ*_1_ in Eq. S7 of Supplementary Note 1, and the initial height *H*_0_ can be obtained by Eq. S3 or S4. The coordinates of each point in the initial configuration are:

$$A\left(0,0,0\right),E\left(R\text{cos}\left(\frac{2\pi }{n}\right),R\text{sin}\left(\frac{2\pi }{n}\right),0\right),....$$

$$\begin{array}{c} B\left(R\text{cos}\theta_{0},R\text{sin}\alpha ,H_{0}\right),\\ D\left(R\text{cos}\left(\frac{2\pi }{n}+\theta_{0}\right),R\text{sin}\left(\frac{2\pi }{n}+\theta_{0}\right),H_{0}\right) \end{array}$$ (2)

### Kinematic analysis model

When the bottom plane of the unit is fixed while a clockwise external torque or a vertical force is applied upwards on the top plane, the unit starts to deform while exhibiting a snapping motion from the initial to folded configurations. This process is a coupled longitudinal and rotational motion of the top plane with respect to the bottom plane. The rotation angle *θ* increases, and the height *h* decreases. The height of the folded configuration is *h*=0, as shown in Fig. 2C.

$$\left|A_{1}B_{1}\right|=2R\text{sin}\frac{\theta }{2}$$ (3)

$$\left|A_{1}B_{2}\right|=2R\text{sin}\left(\frac{\pi }{n}+\frac{\theta }{2}\right)$$ (4)

However, the maximum rotation angle *θ* of the folded configuration is not necessarily equal to *θ*_2_. It is equal to *θ*_2_ only when the initial length of side A_1_B_1_ of the triangle in Eq. 1 is $b=2R\text{sin}\frac{\theta_{2}}{2}$. There are ∠*O*A_1_B_1_ = *β*, ∠ B_1_A_1_B_2_ = *α*, and ∠OA_1_B_2_ = *β* − *α* in Fig. 1C. Thus,

$$\left|A_{1}B_{2}\right|=2R\text{cos}\left(\beta -\alpha \right)$$ (5)

Now, the sides of the triangle in the folded configuration are their initial values |A_1_B_1_| = *b*, |A_1_B_2_| = *c*.

If the initial length of side A_1_B_1_ is $b<2R\text{sin}\frac{\theta_{2}}{2}$, the side length |A_1_B_1_| = *b* is assumed to remain invariant during the deformation. The maximum rotation angle in the folded configuration $\theta_{2}^{\prime }$ is obtained from Eq. 3:

$$\theta_{2}^{\prime }=2a\text{sin}\frac{b}{2R}$$ (6)

During the deformation of the unit, the rotation angle *θ* changes in the range of $\left[\theta_{1},\theta_{2}^{\prime }\right]$. The height *h* and side length |A_1_B_2_| of each state can be determined by Eq. S3 and Eq. 4, respectively, and the coordinates of each point at any instant during deformation can be obtained from Eq. 2.

If the initial length of side A_1_B_1_ is $b<2R\text{sin}\frac{\theta_{2}}{2}$, the side length |A_1_B_2_| = *c* is assumed to remain invariant during the deformation. The maximum rotation angle in the folded configuration $\theta_{2}^{\prime }$ is obtained from Eq. 4:

$$\theta_{2}^{\prime }=2a\text{sin}\frac{c}{2R}-\frac{2\pi }{n}$$ (7)

During deformation of the unit, the rotation angle *θ* changes in the range of $\left[\theta_{1},\theta_{2}^{\prime }\right]$. The height *h* and side length |A_1_B_1_| of each state can be determined by Eq. S4 and Eq. 3, respectively, and the coordinates of each point at any instant during the deformation can be obtained from Eq. 2.

### Quasistatic analysis model

The deformation from the initial to folded configurations is a nonrigid transformation in that, in addition to the local folding of the mountain and valley creases, elastic deformation of the flat panels is needed to complete the transformation. To account for panel bending, the length of crease A_1_, B_1_ is assumed to vary throughout deformation, and a “virtual fold” B_2_V can be created between 2 vertices A_1_, B_1_ across a bending panel A_1_B_1_B_2_, as shown in Fig. 1D [26].

The elastic energy of the unit during deformation is determined through the use of virtual folds and appropriate torsional springs at each crease. The total elastic potential energy of the unit is therefore assumed to take the form

$$\begin{array}{c} \;E=\frac{nk_{1}}{2}\sum_{i=1}^{3}{\left(\gamma_{i}-\gamma_{i0}\right)}^{2}+\frac{nk_{2}}{2}{\left(\gamma_{4}\right)}^{2}\\ \;+\frac{nk_{3}}{4}\sum_{i=1}^{3}\left[1-H\left(\gamma_{i}-\gamma_{\mathrm{ip}}\right)\right]{\left(\gamma_{i}-\gamma_{\mathrm{ip}}\right)}^{4} \end{array}$$ (8)

where the first term is the elastic potential energy of the creases A_1_B_2_, A_2_B_2_, A_1_A_2_. The second term is the elastic potential energy at the virtual crease. The third term is the potential energy of adjacent panel contacts when the unit is close to the folded configuration. $\begin{array}{c} k_{1}=\frac{Et^{3}}{12l} \end{array}$ is the torsional stiffness coefficient of these creases. *γ*_i_ is the angle between the panels shared by a given crease, and *γ*_i0_ is the reference angle of *γ_i_*, which can be obtained in Supplementary Note 2. *k*_2_ is the bending stiffness of the bending panels. *γ*_4_ is the angle at virtual fold crease B_2_V, which can be obtained in Supplementary Note 2. *k*_3_ is the cubic stiffness coefficient. *γ*_ip_ is the threshold of the angles between the 3 types of creases. When *γ*_i_ is less than *γ*_ip_, the panels share the crease contact. $\begin{array}{c} H \end{array}$ is the Heaviside function.

The potential energy as a function of rotation angle *θ* can then be used to determine the torque associated with holding the unit in a certain configuration. According to the principle of energy conservation, the relationship of energy conversion for the unit during the deformation process can be formulated.

During the deformation process, the work done by the pneumatic pressure and the external torque is transformed into the elastic energy of the unit and the output work; hence,

PdV+Tdθ=dE (9)

where *P* and *T* are the pneumatic pressure and the external torque of the unit, respectively. *dV* and *dθ* are differentials of the volume of the inner cavity and rotational displacement. The volume of the inner cavity is formulated in Supplementary Note 2.

Similarly, by derivative of the above equation with respect to *θ*, there is:

$$P\times \frac{\mathrm{dV}\left(\theta \right)}{\mathrm{d\theta}}+T=\frac{\mathrm{dE}\left(\theta \right)}{d\theta }$$ (10)

When the pneumatic pressure and the external torque are known, the rotation angle *θ* can be obtained from the above equation, and then the deformation and configuration of the unit can be determined.

### Structure and material design of soft actuator

**Fig. 3.**
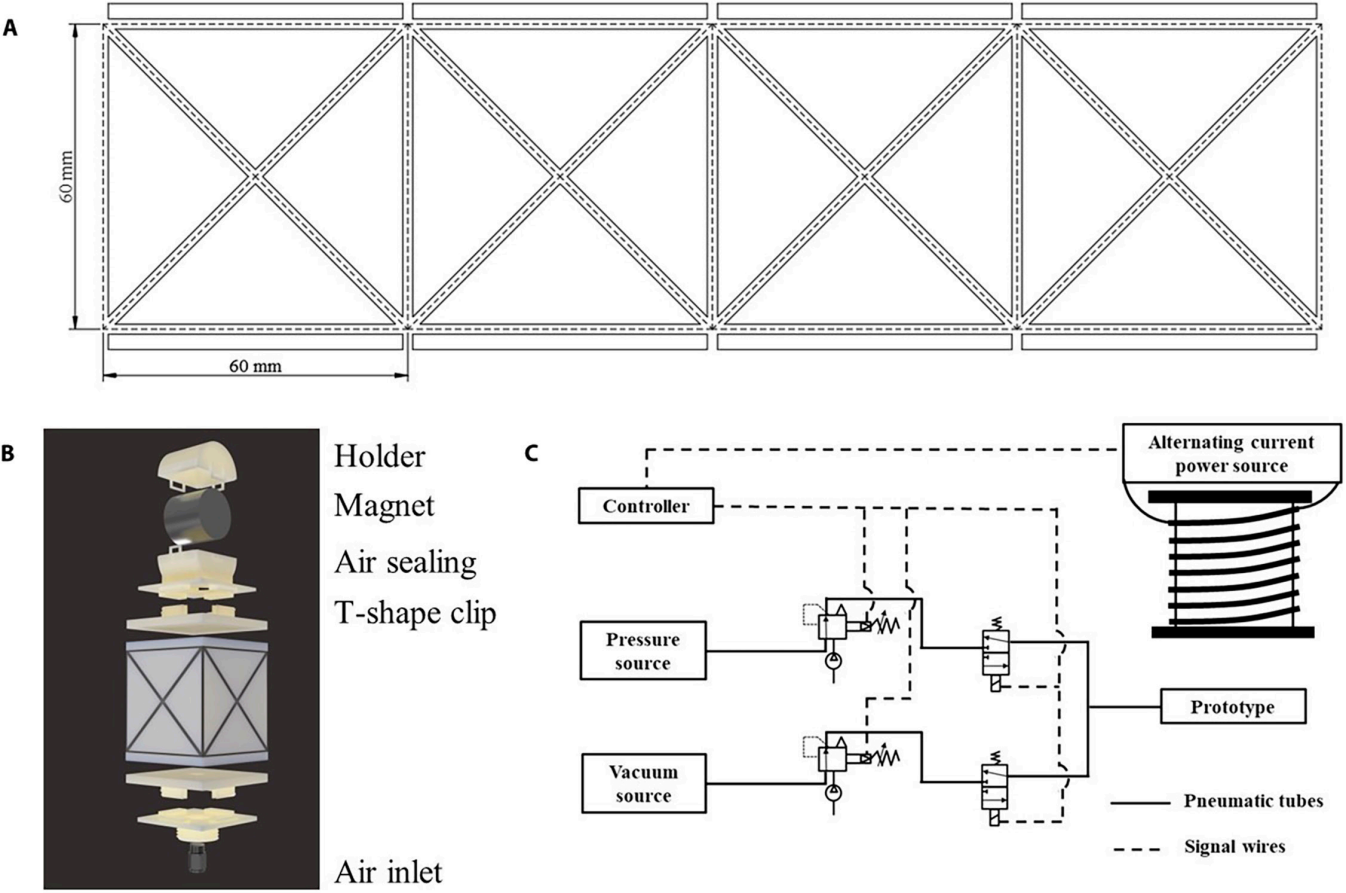
The designed wrist-inspired soft actuator. (A) shows the crease pattern of the origami unit for the wrist-inspired soft actuators shown in (B). (B) shows the soft actuator system, including the holder, magnet, air sealing, T-shaped clip, and air inlet. (C) shows the hybrid driving control system for controlling the external magnetic field and the pneumatic pressure of the wrist-inspired soft actuators.

To achieve bidirectional torsion motion, the crease pattern shown in Fig. 2A is improved by adding a crease in each parallelogram, as shown in Fig. 3A. Now, each parallelogram is bisected into 4 similar triangles. The 4 design parameters are *n* = 4, *a* = 60 mm, *c* = 84.85 mm, and *α* = 45°. The initial height is *h* = 60 mm, and the rotation angle *θ*_0_ = 0°. A wrist-inspired soft actuator is designed based on the crease pattern, as shown in Fig. 3B.

The flat panels are made of polyvinyl chloride (thickness is 0.5 mm), which are cut on adhesive cutting mats using Silhouette CAMEO 4. The PVC panels are then glued to a piece of scotch tape (thickness is 0.13 mm) according to the origami pattern. The soft actuator is formed by combining these structures. The Ecoflex layer, for air sealing, and T-shaped clips are placed on the top and bottom panels of the soft actuator. The air inlet is arranged on the bottom panel. The holder for fixing the magnet is placed on the top panel. The 3D-printed T-shape clips and holder are made of PLA. During repeated folding, the scotch tape used around the fold creases in an actuator is prone to puncturing or fraying, posing a challenge to the durability and reliability of the actuator. To address this issue, silicone coating is applied to the inner wall of the actuator. Additionally, to minimize the friction that occurs in the self-contacting parts of the origami structure, petroleum jelly is applied. By decreasing friction, the deformation process of the actuator becomes smoother and more efficient.

### Magneto-pneumatic hybrid driving method

The initial cross-section of the soft actuator is a quadrilateral. First, an external magnetic field *B* in the plane of the top panel is applied to the actuator, which results in an in-plane torque *T* = *V*(*M* × *B*), where *M* is the magnetization and *V* is the volume of the magnet. The initial angle between *M* and *B* is 90°. The top panel driven by magnetic torque will twist at a certain angle. Then, the inner cavity of the actuator is vacuumed. The actuator compresses and twists under the combined action of vacuum pressure and a magnetic field until it is completely folded. Remove the external magnetic field and inflate the actuator, and return to the initial configuration. Then, a magnetic field in the opposite direction is applied to the actuator, and the top panel driven by magnetic torque will twist in the opposite direction until it is completely folded. Finally, the actuator will return to the initial configuration under the action of vacuum pressure. Thus, the actuator completes a periodic deformation process.

The bidirectional torsion of the actuator is achieved by regulating the direction of the external magnetic field. Obviously, the external magnetic field strength and pneumatic pressure also need to be precisely controlled. The magneto-pneumatic hybrid driving control system is shown in Fig. 2C. The pneumatic control system consists of 2 parts: the pneumatic supply and control systems. The positive pressure was provided by a pressure pump (OUTSTANDING, Inc., China), and the vacuum was provided by a vacuum pump (550D, Fujiwara). Solenoid valves KLC2-A (Kamoer Inc.) is used to control the inflatable pressure and the vacuum pressure. The solenoid valve is located between the pumps and actuator and is connected with them through pneumatic tubes. The switches of the solenoid valves are controlled by the signal from the microcontroller (Arduino mega 2560, Arduino Inc.) via relays (TOUGLESY, Batu Na Group Co., Limited) powered by a 24-V power supply. The external magnetic field was provided by an electromagnet KK-P200/40 (KAKCOM Inc.), and the direction and strength of the external magnetic field are controlled by the signal from the microcontroller (Arduino mega 2560, Arduino Inc.). A laptop (Y9000X, Lenovo, Inc., China) is used to run the Proportional-Integral-Derivative (PID) control algorithm. A pressure sensor (ZSE30AF-01-E, SMC, Inc., China) is used to measure pneumatic pressure and a gaussmeter (TM5100, TUNKIA, Inc., China) is used to measure external magnetic field.

### Results and DiscussionKinematic analysis

**Fig. 4.**
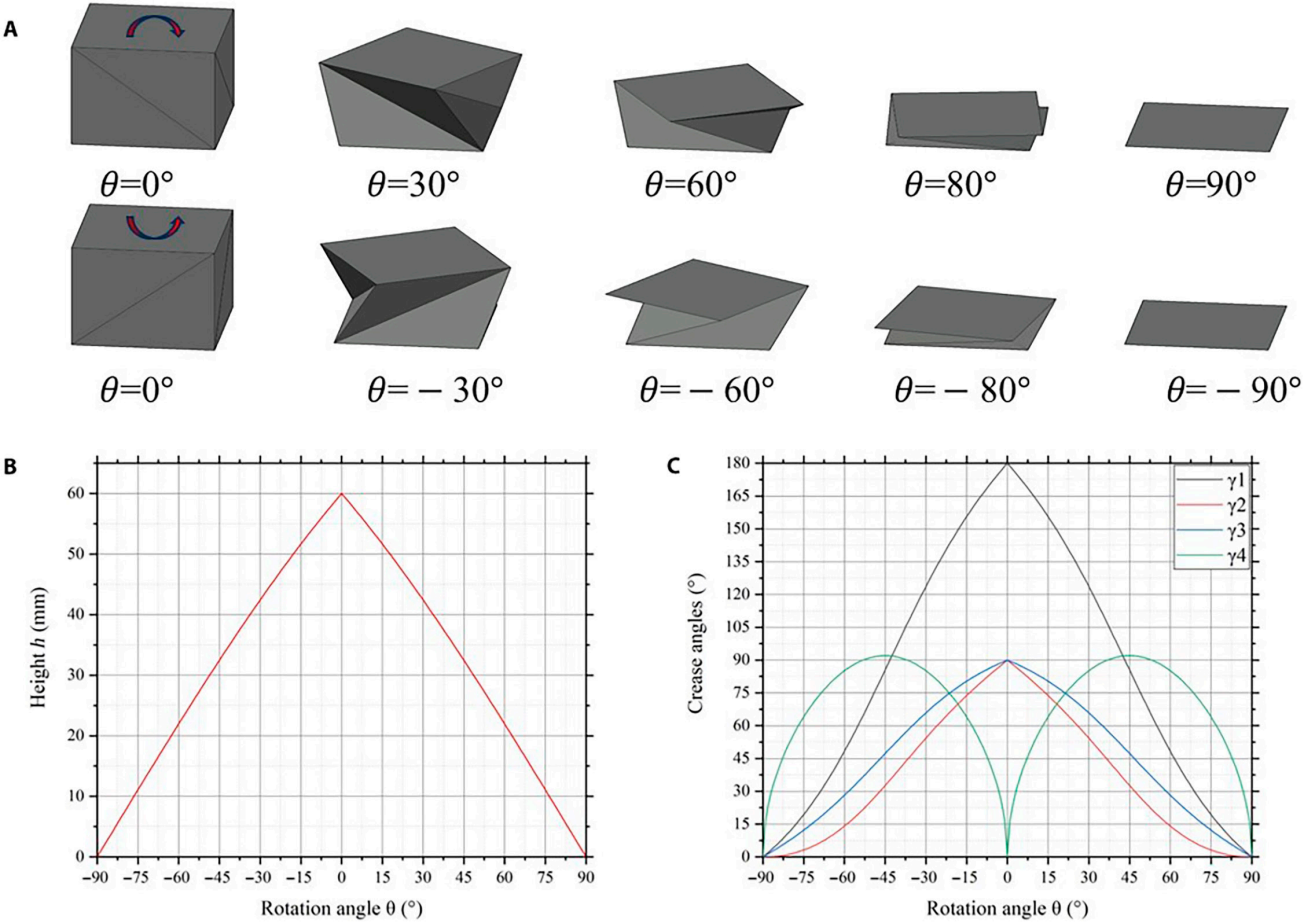
Kinematic properties of the soft actuator. (A) Bidirectional torsion deformation process of the soft actuator. (B) Height *h* of the soft actuator plotted as a function of rotation angle *θ*. (C) Crease angles *γ*_1_,*γ*_2_,*γ*_3_,*γ*_4_ plotted as a function of rotation angle *θ*.

The deformation process of the soft actuator can be obtained by using the abovementioned kinematic analysis model, as shown in Fig. 4A. The rotation angle is *θ* = 0° in the initial configuration. Here, we define the rotation angle as a positive value when the actuator rotates clockwise. When a clockwise magnetic moment is applied to the top panel, the actuator twists clockwise and compresses. The actuator is fully folded when *θ* = 90°. Similarly, when an anticlockwise magnetic moment is applied to the top panel, the actuator twists anticlockwise and compresses. The actuator is fully folded when *θ* = −90°. The figure shows that the actuator can achieve bidirectional torsion deformation and can be fully folded.

Figure 4B shows the change in height of the actuator during bidirectional torsion. The height of the actuator changes substantially with the continuous increase in rotation angle *θ*. The changes in crease angles γ_1_, γ_2_, γ_3_ and virtual crease angles γ_4_ with rotation angles *θ* are shown in Fig. 4C. It can be seen from the figure that the creases A_1_B_2_ are flat in the initial configuration and folded in half when the actuator is fully folded. The crease angle of A_2_B_2_, A_1_A_2_ is 90° in the initial configuration and is completely pressed when the actuator is fully folded. The virtual crease B_2_V is flat in the initial configuration and reaches a maximum value of 92° when the rotation angle is *θ* = 44°. The virtual crease B_2_V is fully opened again when the actuator is fully folded. This means that the flat panels have a large bending deformation during the deformation process of the actuator.

### Quasistatic characteristics experiments

Numerous experiments have been carried out to showcase the quasistatic characteristics of the soft actuator. These experiments include compression experiment, actuation experiment, and fatigue characteristic experiments.

**Fig. 5.**
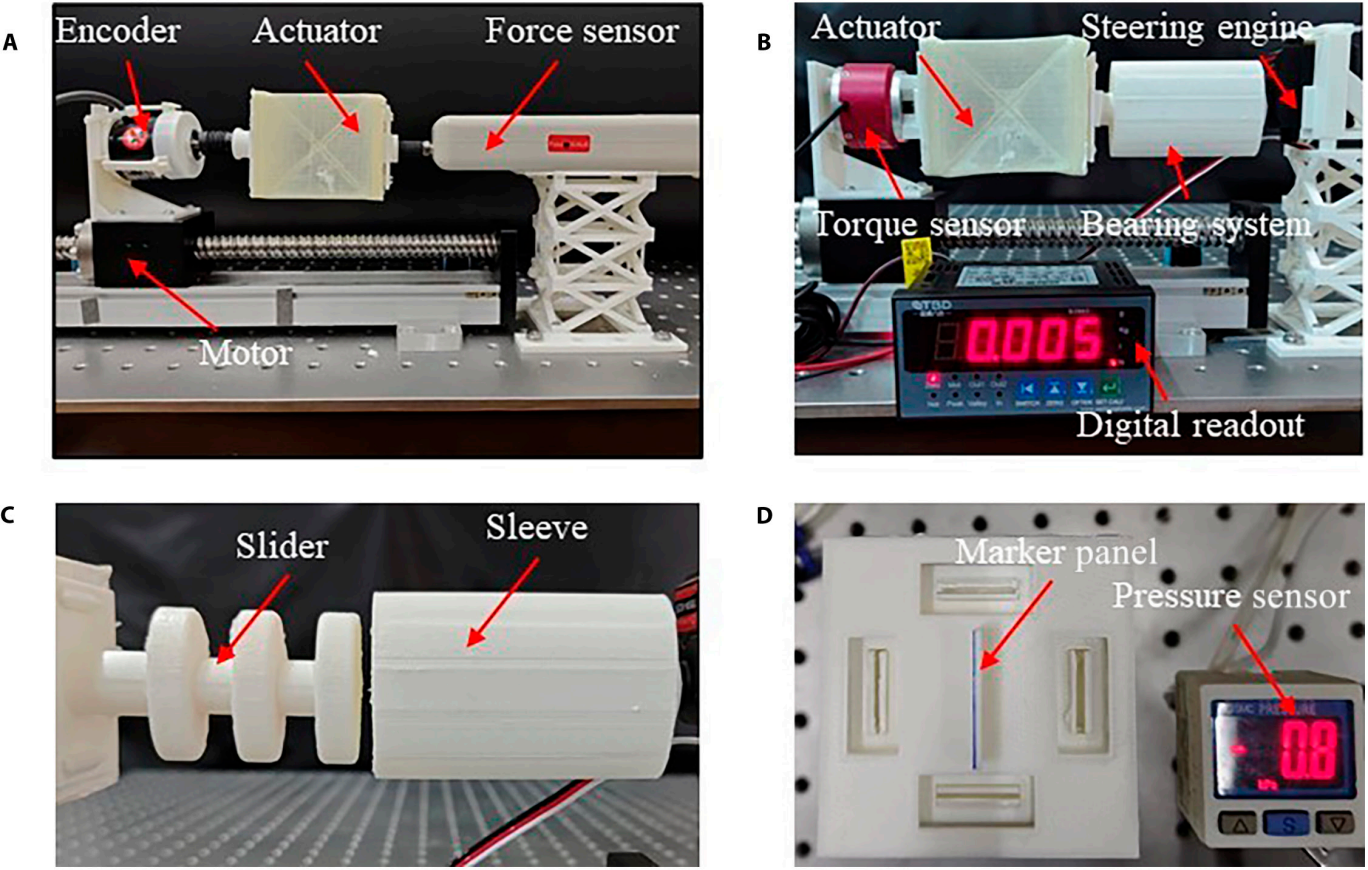
Experimental devices for the soft actuator. (A) Device of displacement versus force. (B) Devices of displacement versus torque, where a thrust bearing is applied to allow the rotary bearing to rotate freely. (C) The bearing system used in (B). (D) Device for obtaining the pressure–deformation relationship.

**Fig. 6.**
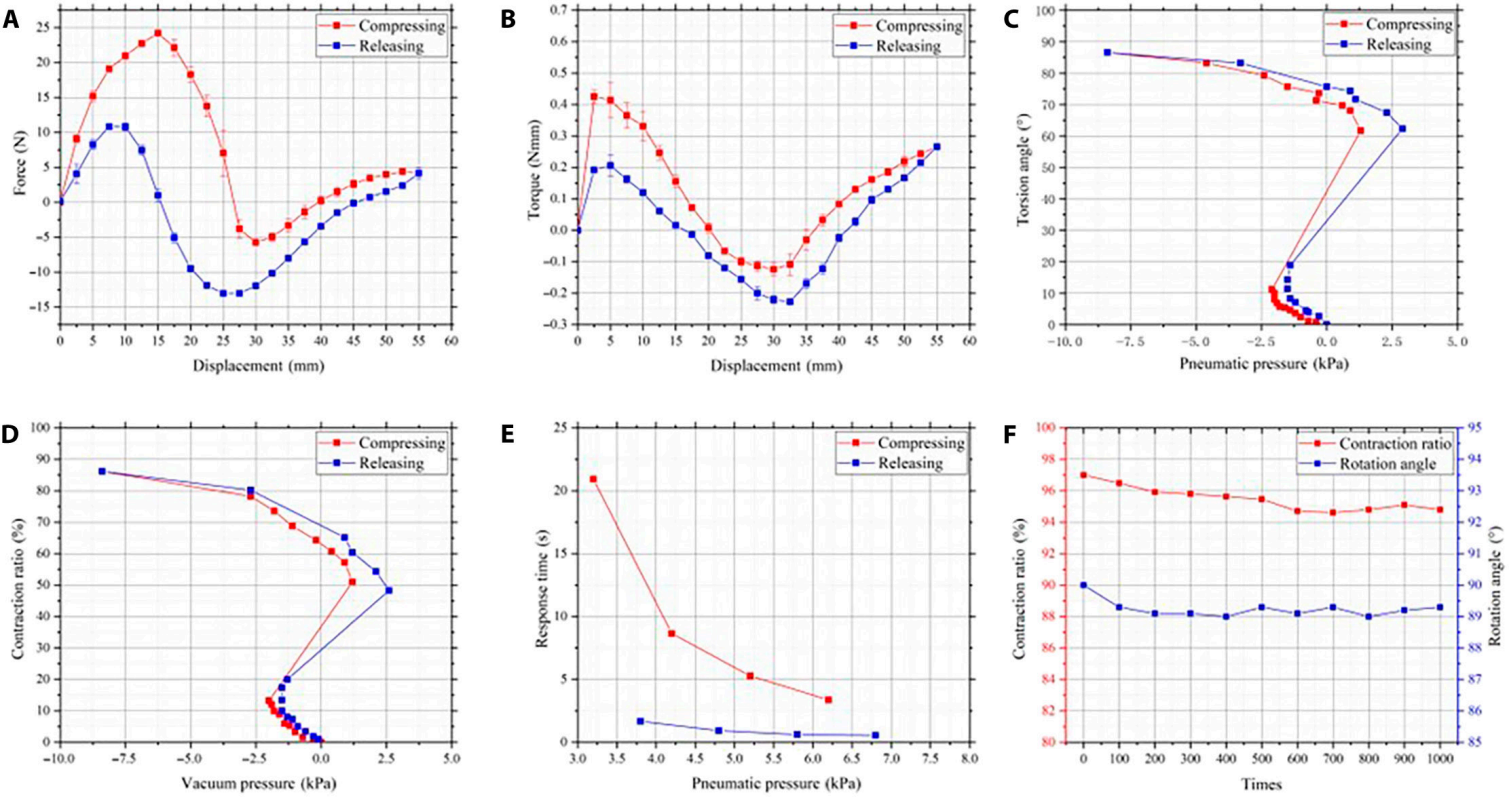
Quasistatic characteristics verification of the soft actuator. (A and B) Diagrams for displacement–force and displacement–torque. (C and D) Diagrams for pressure–angle and pressure–contraction relationships. (E) Response time for both compressing and releasing processes under various pneumatic pressures. (F) Deformation properties of fatigue characteristic experiments.

To investigate the mechanical properties of the soft actuator, specifically its behaviors under compression, a compression experiment is conducted to obtain the relationship between displacement and 2 key parameters: force and torque. A compression device equipped with a feeding mechanism and various sensors including force sensor (DS2-500N, ZHIQU, Inc., China), rotary encoder (E6B2-C2Z6C, OMRON), and torque sensor (S4002, STBD) is used for this purpose. The soft actuator is securely fixed on the base and the feeding mechanism controls its deformation, as illustrated in Fig. 5A and B. For precise control of the deformation, the motor controller is used to give 1,600 pulses to drive the stepper motor, resulting in a 36° rotation. This motion, in turn, moves a high-precision screw (with a resolution of 0.02 mm), pushing the slider by 1 mm. Therefore, the number of pulses serves as a measure for calculating the actuator’s displacement. The compression device compresses the soft actuator at a controlled speed of 1.67 mm/s (as shown in Fig. 5A) and then releases it, repeating this process 3 times. The relationship between displacement and force can be found in Fig. 6A and the relationship between displacement and torque is shown in Fig. 6B. Notably, the force or torque during the releasing phase is observed to be smaller than during compressing phase, likely due to energy consumption. However, both the compressing and releasing phases exhibit the same bistable characteristics of the soft actuator, highlighting its unique mechanical properties.

To assess the basic actuating properties of the soft actuator, such as rotational angle and contraction ratio, it is subjected to an actuation experiment under varying pneumatic pressures. As shown in Fig. 5D, a marker panel is mounted on the top surface of the soft actuator. The deformation of the actuator is recorded by a camera and analyzed in Tracker 4.91 software. During the experiment, the vacuum regulator is carefully adjusted to decrease the vacuum pressure. The deformation of the actuator is measured after a stabilization period of approximately 20 s. To examine the releasing process of the actuator, it is subsequently inflated after being fully folded. Figure 6C and D illustrate both the pressure–displacement and pressure–angle relationship. A key finding from these results is the observation of an abrupt process in the pressure and pressure–contraction data. This phenomenon is attributed to the bistable nature of the actuator, which exhibits snap-through behavior between its 2 stable states. A significant outcome of the experiment is the demonstration of the actuator’s ability to achieve a high contraction ratio and substantial rotation angle. Specifically, under a vacuum pressure of 8.4 kPa, the actuator attains a contraction ratio of 86.22% and outputs a rotation angle of 86.6°. These findings highlight the actuator’s efficiency and effectiveness in transforming pneumatic pressure into torsion motion.

To evaluate the response time of the soft actuator under different conditions, an actuation experiment is conducted using varying pneumatic pressures for compression and release cycles. The deformation of the soft actuator is recorded using a camera capable of capturing 30 frames per second. By analyzing the motion information captured in these recordings, the time taken for both compressing and releasing the actuator is accurately determined. As illustrated in Fig. 6E, there is a notable positive correlation between the response time of the actuator and the applied pneumatic pressure. Specifically, the results indicate that under a vacuum pressure of −6.2 kPa, the actuator takes approximately 3.77 s to compress. Conversely, during the release phase with a pressure of 6.8 kPa, the actuator takes about 0.55 s to return to its initial configuration.

The fatigue characteristics of the soft actuator are experimentally assessed through fatigue characteristics experiments, as shown in Fig. 5D. The experiment is essential to determine the actuator’s endurance and reliability over extended periods of use. During the experiment, the soft actuator undergoes a specific pneumatic pressure cycle: it is deformed under a pressure of −22 kPa and then returned to its initial configuration with a pressure of 24 kPa. The timings are carefully controlled, with 1.0 s allocated for the compression phase (to ensure complete folding) and 0.7 s for the releasing phase (to ensure it returns to its initial configuration). A notable finding from these experiments is that the contraction ratio and rotation angle do not continually increase with the number of tests conducted. This observation suggests that the actuator does not experience significant degradation in its capacity to deform, even after numerous cycles. The result in Fig. 6F indicates that the deformation capacities of the soft actuator remained reliable even after 1,000 times repeated operations. However, the experiments also reveal that the primary area of wear or damage tends to be concentrated around the virtual fold crease B_2_V due to the elastic deformation of the flat panels A_1_B_1_B_2_.

**Fig. 7.**
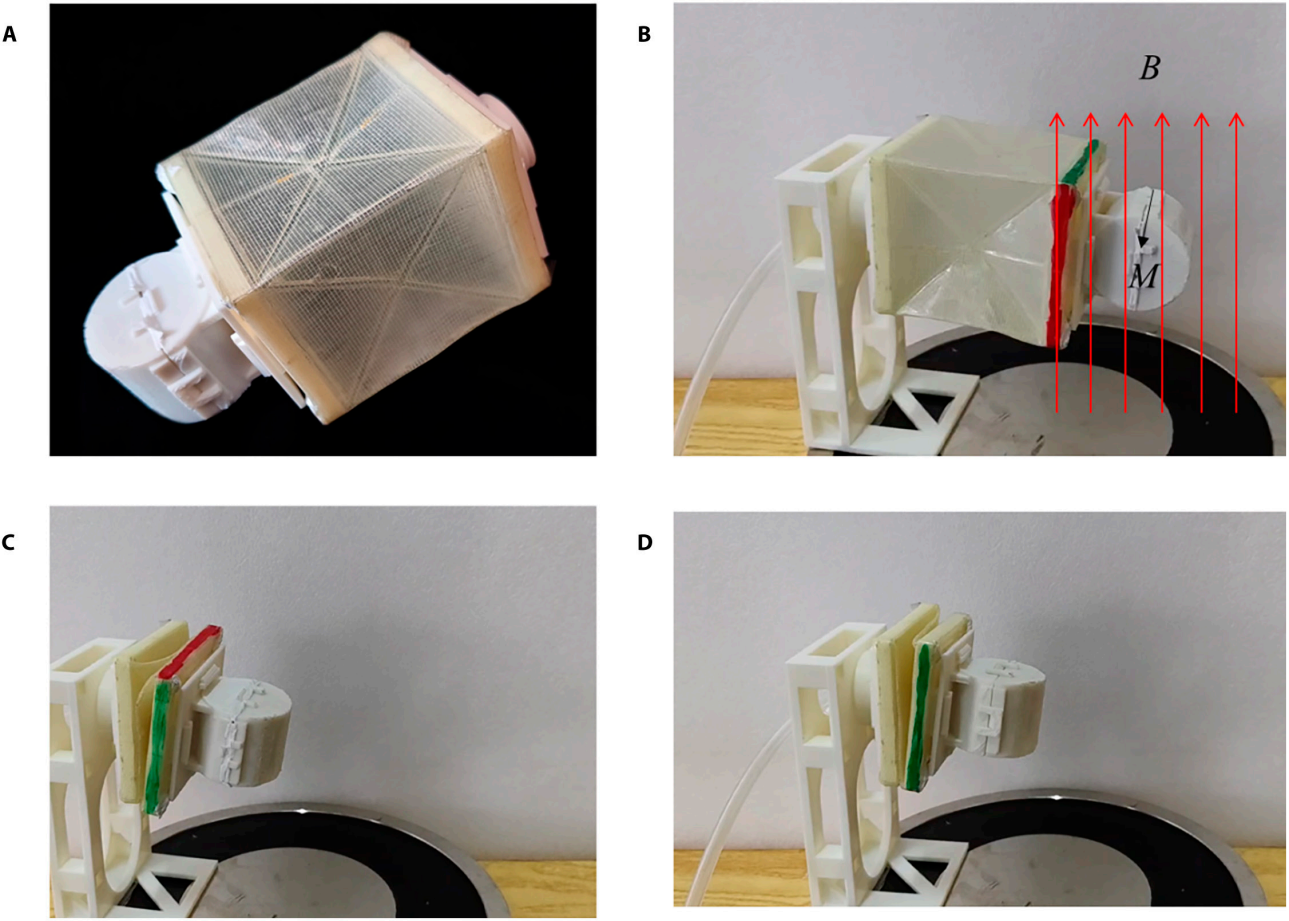
Experimental verification. (A) shows a demo model of the soft actuator. (B) shows the experimental platform to verify the performance of the soft actuator. The left end of the actuator is fixed, and the right end is free. Under the control of the magneto-pneumatic hybrid driving control system, the soft actuator realizes bidirectional torsion. (C) shows the soft actuator fully folded after a clockwise twist deformation. (D) shows the soft actuator fully folded after an anticlockwise twist deformation.

To experimentally verify the magneto-pneumatic hybrid actuation of the soft actuator, a model is manufactured and assembled, as shown in Fig. 7A. The bottom polygon panel of the actuator is fixed, and the top polygon panel is free. An external magnetic field *B* = 6.33 mT is applied through an electromagnet, and the magnetic torque *T* is 0.048 Nm in the initial state. The direction of the magnetic field can be easily manipulated, as shown in Fig. 7B. The soft actuator deforms under external magnetic field and pneumatic pressure, as shown in Movie S1. It can be seen from the experimental results that the actuator has bidirectional torsion deformation ability. When the soft actuator is fully folded after a clockwise or anticlockwise twist deformation, as shown in Fig. 7C and D, it is able to maintain its current states after removing all external stimuli. This verifies that the actuator has 3 steady states and that the rotation angle can vary from −90° to 90°. Its rotation ratio RDH (= rotation angle * diameter of section / height) is more than 254°. The actuator model has a rotation angle range that is the same as that of the human wrist.

**Fig. 8.**
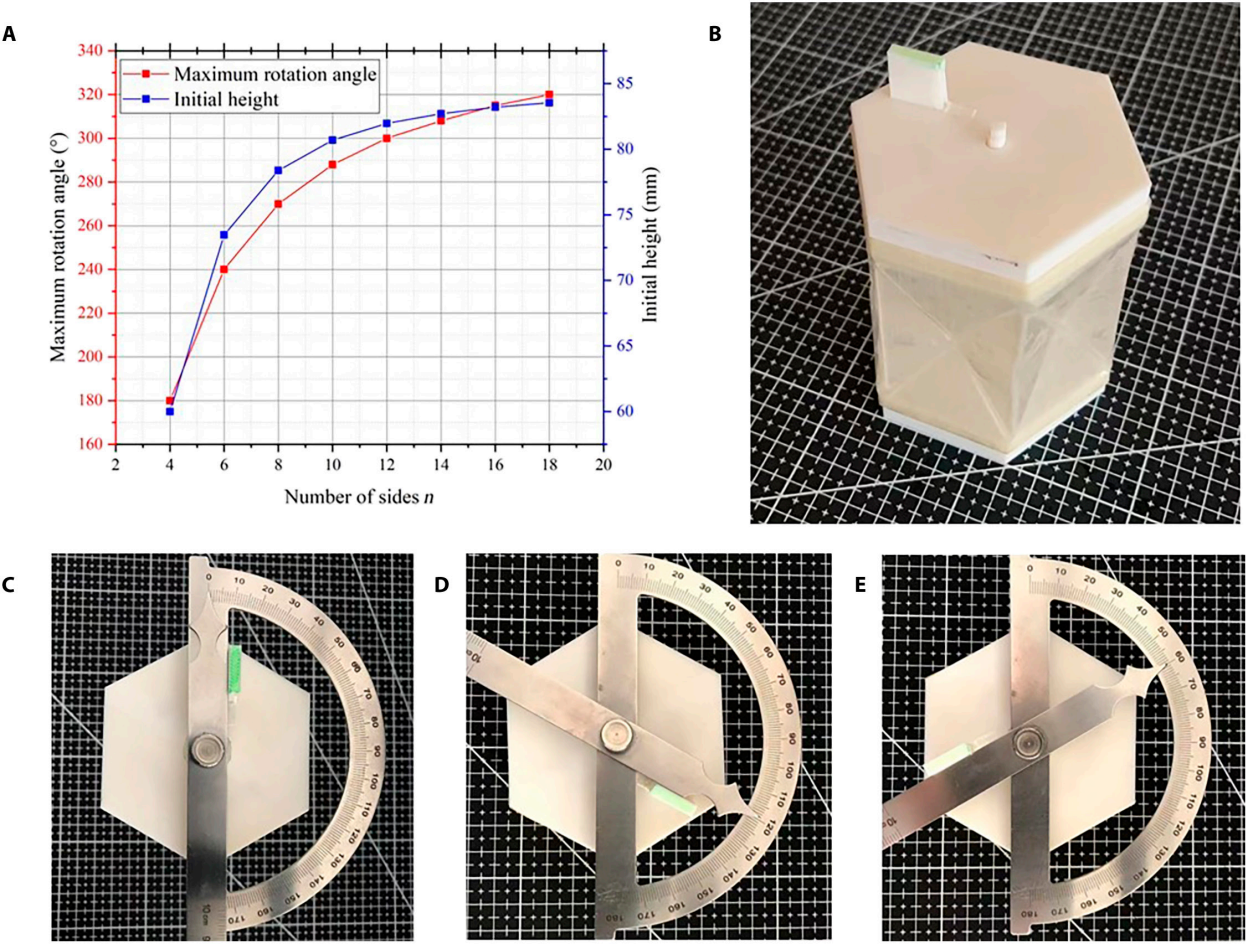
Soft actuator with a larger rotation angle range. (A) Maximum rotation angle and initial height of the actuator as a function of the number of sides *n*. (B) represents the soft actuator with the number of sides *n* = 6, the maximum rotation angle of which is *θ* = 240°. (C) to (E) represent rotation angle measurement of the soft actuator.

### Actuator with larger torsion deformation

To simulate the human wrist, the soft actuator is designed to have a rotation angle range *θ* = 180° in this paper. If needed, we could design a soft actuator with a larger rotation angle range based on the Kresling origami unit. The radius of the outer circle of the section would remain invariable, and the number of sides *n* of the section polygon would be changed. The maximum rotation angle and initial height of the actuator as a function of the number of sides *n* are obtained by using the abovementioned kinematic analysis model, as shown in Fig. 8A. As the number of sides *n* increases, the maximum rotation angle and initial height increase rapidly and then change slowly. When the number of sides *n* is designed to be 18, the maximum rotation angle reaches *θ* = 320°. Now, the initial height of the actuator is *h* = 83.54 mm. Its rotation ratio RDH (= rotation angle * diameter of section / height) is more than 325°.

To corroborate the analytical model and its results, a practical demonstration is conducted using a fabricated twisting actuator with 6 sides (*n* = 6), as illustrated in Fig. 8B. This actuator is designed to twist both clockwise and anticlockwise in response to pneumatic pressure. The key focus of this validation process is to accurately measure the rotation angles of the actuator when it is fully folded. The rotation angles are measured by a protractor (SYNTEK, Inc., China). The procedure for this measurement is detailed in Fig. 8C to E. The experimental results show that the rotation angle of the actuator varies from −120° to 119.5°. The maximum rotation angle of the twisting actuator is *θ* = 239.5°, which is in excellent agreement with the analytical result of *θ* = 240°. Additionally, its rotation ratio RDH (= rotation angle * diameter of section / height) exceeds 277°. Then, the analytical model is confirmed by the experimental results of 2 prototypes (*n* = 4 and *n* = 6).

## Conclusion

A wrist-inspired magneto-pneumatic hybrid-driven soft actuator with bidirectional torsion capabilities based on the Kresling origami unit is studied. The primary objective is to maximize the rotation angle achievable by a single actuator. A unique feature of this actuator is its ability to control the direction of torsion motion through manipulation of the external magnetic field’s direction. To comprehensively understand and predict the behavior of this actuator, both kinematic and quasistatic mechanical analysis models are developed. These models facilitate a detailed examination of the actuator’s mechanical properties. To validate the theoretical models and to demonstrate the practical feasibility of the actuator, a demo model is constructed and tested. The experiment is designed to specifically verify the actuator’s capability for bidirectional torsion motion. The analysis and experimental findings confirm that the actuator possesses 3 steady states and can achieve a rotation angle ranging from −90° to 90°. The actuator model has a rotation angle range that is the same as that of the human wrist. Further exploration into the actuator’s design reveals that by increasing the number of sides (*n*) of the section polygon, an actuator capable of achieving even larger rotation angles can be developed. A twisting actuator with the number of sides *n* = 6 achieves a rotation angle of 239.5° and its rotation ratio (=rotation angle/aspect ratio) is more than 277°, about twice the largest one in other literature. Future research will focus on optimizing its load capacity, further enhancing its applicability and efficiency in various robotic and mechanical systems.

## Data Availability

All data needed to evaluate the conclusions in the paper are present in the paper and/or the Supplementary Materials. Additional data related to this paper may be requested from the authors.
